# The Effects of a Child-Focused Coping Intervention on Parental Coping and Parent Depressive Symptoms in a Randomized Control Trial

**DOI:** 10.1007/s10826-025-03235-4

**Published:** 2025-12-22

**Authors:** Adithi Rajagopalan, Martha Wadsworth

**Affiliations:** Department of Psychology, The Pennsylvania State University, University Park, PA USA

**Keywords:** Coping, Adolescence, Caregiver outcomes, Parent coping, Coping intervention, Depression

## Abstract

Poverty and its related stressors have been shown to lead to poor mental and physical health outcomes for entire family systems including youth and their caregivers. However, active and engagement coping strategies have been shown to disrupt this relationship, protecting against negative outcomes for youth and their caregivers. The Adaptation to Poverty Related Stress model posits that coping for children and caregivers is related in a dyadic nature, such that the ability of each unit to engage useful coping mechanisms impacts the others. Research has primarily focused on the impact of parent-focused interventions on child coping. Little research has explored how child-focused interventions impact parent coping and parent mental health. The Building a Strong Identity and Coping Skills Intervention (BaSICS), a child-focused coping intervention, was designed to educate youth about multiple uncontrollable stressors and promote coping skills development. This intervention has been shown to improve child mental health. This study explored the mediating nature of parent coping following a child’s enrollment in BaSICS on parent depressive symptoms during a randomized control trial (RCT). Results revealed a mediating relationship of parent primary control coping. Additional hierarchical regressions demonstrated that parent secondary control coping was promotive of a reduction in parent depressive symptoms, as was child use of problem-solving. This study demonstrated that child coping, whether through modeling or other mechanisms, can positively impact parent coping and mental health. This suggests that child-focused programming may be another point of intervention for families in high-stress contexts.

## Highlights

Parents of children who participated in the youth-focused BaSICS coping and empowerment program had significant decreases in depressive symptoms over time.

Parents of children in BaSICS showed improved primary control coping (problem solving, emotion regulation) over time, relative to parents of control youth.

Changes in parent primary control coping mediated the relationship between child participation in BaSICS and parent-reported depressive symptoms.

Poverty, economic disadvantage, and related family stressors are associated with poor mental and physical health outcomes in both youth and their caregivers. Research has demonstrated a multitude of pathways that may lead to these poor mental and physical health outcomes for families in poverty. Among the most pervasive and powerful pathways is chronic and uncontrollable poverty-related stress (PRS) (Reiss, 2013; Wadsworth, 2015), a term developed by Wadsworth & Berger (2006), to comprise all associated stress due to poverty (e.g. housing instability, socioeconomic strain, lack of resources). Coping, or regulatory processes aimed at managing cognitive, emotional and physiological reactivity in response to stress, has been shown to ameliorate and protect against these negative outcomes in both children and parents (Compas et al., 2001). Engagement coping strategies, defined as active methods of coping such as problem solving and emotion regulation, in particular have been shown to be useful in the face of uncontrollable stressors (Perzow et al., 2021). However, research has demonstrated that exposure to high levels of stress can interfere with the ability to engage in typically efficacious engagement coping.

Poverty has been shown to impact parental mental health due to the exposure to economic hardship, and associated PRS (Wadsworth, Raviv, Santiago & Etter, 2011). Chronic financial stress has been shown to be associated with greater family conflict, interparental discord (Conger & Conger, 2002; Conger et al., 2002), unsuitable housing (e.g. exposure to toxins), increased adolescent mental health difficulties including internalizing symptoms, negative emotionality, attention and cognitive deficits (De France, Stack & Serbin, 2023) and decreased parental ability to effectively manage and cope with stress. The Adaptation to Poverty Related Stress (APRS) model has demonstrated that these stressors both directly and indirectly increase parental depressive symptoms, with coping being a primary mechanism. More specifically, as reported stress increases, parents’ ability to cope decreases, leading to worsened mental health outcomes (Wadsworth et al., 2011).

As proposed by the Adaptation to Poverty Related Stress (APRS) model, and demonstrated by Wadsworth & colleagues (2011), parental coping and child coping are related in a dyadic, bidirectional manner such that a child’s ability to engage available coping strategies is associated with a parent’s coping engagement. When caregivers are better able to cope with stress, children are better able to cope with stress, and, relatedly, when children are better able to cope with stress, caregivers are better able to cope with stress. Furthermore, previous research has shown that engagement coping in youth appeared to mitigate the adverse effects of poverty related stress on internalizing symptoms for both the adolescents themselves and their parents in a sample of low-income families (Wadsworth et al., 2011). Hence, it is possible that improving adolescents’ ability to cope with poverty-related stress could have positive spillover effects on parents—reducing their stress, improving their coping, and reducing depressive symptoms.

As of yet, research has primarily focused on the relationships between poverty-related stress, caregiver coping, and child coping. For example, previous research has examined the ways in which caregiver coping relates to child coping, finding that, caregivers’ reactions to stress and their coping responses significantly predicts child coping ability (Hakim-Larson, 1999; Valiente et al., 2004). These findings are consistent with the APRS’ modeling of the bidirectional nature of caregiver-child coping. In a study of diverse families enrolled in a parenting program, Cappa & colleagues (2011), found a reciprocal relationship between caregiver and child stress and coping. They found that children whose caregiver reported greater stress tended to have more difficulty effectively coping with challenges such as academic and social responsibilities. These findings suggest that parent stress levels can impact child coping skills and well-being. However, research examining how child coping impacts parent coping and well-being remains lacking. To our knowledge, no studies have examined the reverse effect, that is, how child participation in a coping intervention influences parent coping and well-being.

The Building a Strong Identity and Coping Skills (BaSICS) program, which was developed specifically to improve young adolescents’ ability to cope effectively with poverty-related stress, has demonstrated significant and sustained increases in adolescent engagement coping (Wadsworth et al., 2022, 2015, 2011). Given the bidirectionality of parent and child coping, might successful coping acquisition of adolescents translate into improved coping and outcomes for parents? The present study was designed to test whether the child-centered coping BaSICS intervention has a positive impact on caregiver coping and psychological outcomes. Specifically, the present study examined whether a child’s participation in BaSICS improves caregiver use of coping strategies, which in turn reduces depressive symptoms.

## Targeting Early Adolescence

Early adolescence constitutes an ideal development window for coping-focused intervention. This is a time characterized by increased neuroplasticity and the capacity to learn and develop new coping skills that have the opportunity to significantly improve long-term psychosocial and health outcomes. It is likely that adolescents exposed to chronic and uncontrollable stress display significantly different biological (e.g., cortisol reactivity, HPA engagement) and psychological responses to stress than adolescents who do not have similar exposures (Wadsworth, 2022; Pham & Bendezu, 2023). This elevated reactivity is linked with negative long term physical and mental health outcomes. However, during puberty, adolescents rapidly develop behavioral and emotional capabilities necessarily to engage more sophisticated coping skills (Skinner, Zimmer-Gembeck, 2007). Harnessing this sensitive period and helping adolescents develop a stronger and more balanced coping repertoire has been shown by (Pham et al., 2023) to decrease cortisol hyperreactivity.

## Parent Mental Health

Extensive research has shown associations between parent mental illness and child mental illness, including for example, depression and anxiety as well as personality disorders and schizophrenia (Beardslee, Gladstone & O’Connor, 2011; van Santvoort et al., 2015; Hameed & Lewis, 2016). Parental mental illness has also been associated with adverse physical and mental health outcomes, parent-child interaction difficulties, challenges in either developing or utilizing appropriate parenting skills, which then are also conversely associated with greater parental mental health difficulties and subsequent child mental health difficulties (Barnow et al., 2006). However, the engagement of coping skills for parents has been repeatedly demonstrated to help mitigate the effects of parent mental illness on both members of the parent-child dyad. Engagement coping, such as problem-solving and emotion-focused coping skills, social support seeking, and cognitive restructuring have been shown to be associated with fewer endorsed mental health challenges in both parents and children (Hosman, van Doesum, van Santvoort, 2009; Sell et al., 2021). However, the inverse relationship (i.e. child coping and parent mental health and coping engagement) has not been demonstrated in research involving child-focused interventions or the context of poverty related stress.

## Current Study

Enrollment in the child-focused coping intervention, BaSICS, has been shown to improve child psychosocial functioning and increase a child’s ability to engage in helpful engagement coping skills, such as problem-solving (Raviv and Wadsworth, 2010; Wadsworth et al., 2013; Wadsworth et al., 2018). (McDonald et al., 2020; Pham et al., 2023) found that adolescents who participated in this short-term program aimed at teaching primary and secondary control coping, and improving a adolescents coping repertoire had an improvement in multiple facets of well-being including greater use of problem-solving and cognitive restructuring skills (i.e., problem-solving steps Raviv and Wadsworth, 2011), a significant reduction in avoidant coping strategies, and decreased HPA reactivity, as compared to adolescents not enrolled in the intervention. Additionally, this same intervention was shown to improve child internalizing and somatic symptoms, by both parent and child report. Pham and colleagues (2023) further identified that these improvements were still apparent longitudinally, one-year post-intervention. However, these studies did not address whether these improvements in coping repertoire, internalizing symptoms, and HPA reactivity, elicited improvements in parent mental health and coping.

Existing literature does not address whether interventions focused on adolescents’ coping will impact, and possibly improve, parents’ mental health. This current study aims to address this gap, testing the impact of a child’s enrollment in a psychoeducational coping intervention on parents’ depressive symptoms with parent coping as a mediator. Little is understood regarding the relationship between child and parent coping and mental health and the mechanisms by which these relationships may be interconnected. However, children learning coping strategies may choose to teach their parents new skills that they have learned, thus increasing parents’ repertoire. Understanding the pathways that might lead to improved parent mental health could improve our ability to implement interventions and increase our understanding of contextual coping. This study, then, seeks to understand how an intervention targeting child coping might improve parents’ ability to tolerate and cope with, uncontrollable stressors such as poverty-related stress, thus resulting in a decrease in depressive symptoms. We hypothesize that enrollment in an educational coping intervention for adolescents (BaSICS) will be associated with an increase in parent-used primary control coping, as these strategies, such as problem-solving, may be easier for adolescents to actively demonstrate and teach to their parents. This increase in parent primary control coping will then be associated with a decrease in parent-reported depressive symptoms.

## Method

### Participants

Parent-child dyads were recruited from two urban neighborhoods in central Pennsylvania, both with high reported crime (i.e., trauma, community violence) where the violent crime rate in both neighborhoods is more than 250% US average. The neighborhoods have high rates of poverty with 40–44% of children under age 18 living below the poverty line (U.S. Census Bureau, 2018). The U.S. Census Bureau reports that the largest identified racial/ethnic group of both neighborhoods was African American (43% parents/52% children), followed by White (45%/39%) and Hispanic Latino (17%/20%).

Eleven and twelve-year-old children who did not report clinically elevated anxiety or depression, evidence of an autism spectrum disorder or intellectual disability were eligible. Additionally, families of participants must have had an income of <=200% FPL, and at least one parent or guardian who agreed to complete the research study. Twenty four of the 183 adolescents who completed eligibility assessments were not eligible for the study due to not meeting the previous criteria, and 68 adolescents who were eligible declined to participate.

**Fig. 1:**
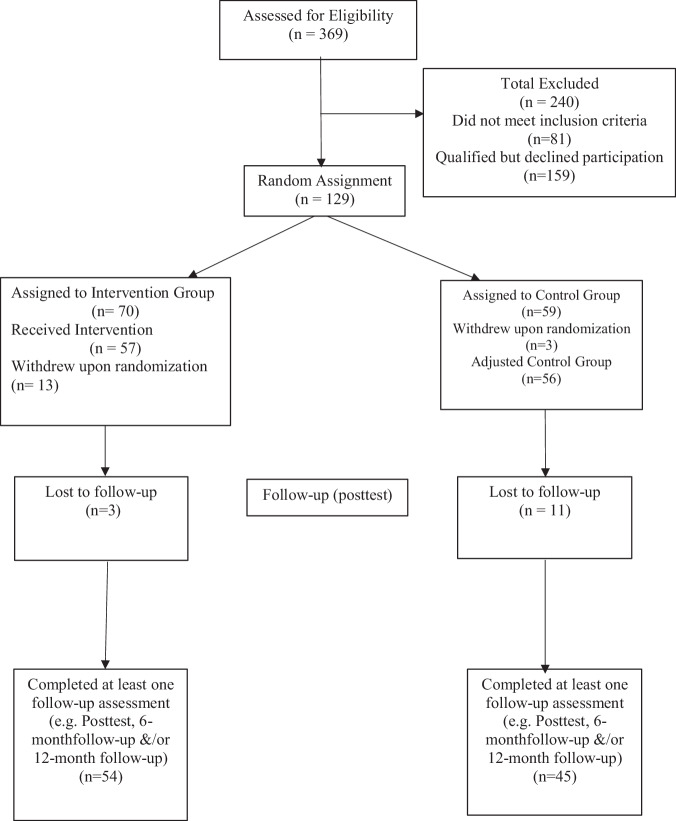
Consort Table

One-hundred and twenty-nine parents and children (40% of children and 6% of caregivers were male) were recruited into the study and completed pre-test measures (Fig. 1). Twelve dyads withdrew prior to the start of the intervention, with a final sample of *N* = 117. There were no significant differences between the dropped participants and those remaining in the study on measures of child internalizing, parental depression, or perceived economic hardship. All families who left the study reported that they could no longer participate in the intervention due to scheduling difficulties. All data reported in tables and text were limited to the analysis sample. Children were an average age of 11.81 years old (*SD* = 0.55) and were 45% Hispanic/Latino, 55% Non-Hispanic Black, 17% Non-Hispanic White, and 28% Other/Multiracial. Parent age ranged from 26–63 years of age (*M* = 39.37, *SD* = 8.28), and parents were 34.9% Hispanic/Latino, 51% Non-Hispanic Black, 21% Non-Hispanic White, and 28% Other/Multiracial. According to the U.S. Census Bureau, the 2018 federal poverty threshold for a family of four was set at $28,780 (2017). The average household income for participants was $24,422 (*SD* = 32,396). The majority of parents were employed (57% were either employed full-time or part-time), 21% were unemployed, 13% were on disability and 9% were homemakers, full-time students, or retired. 30% of parents did not complete high school, 27% had a high school diploma or GED, and 43% had schooling beyond high school. Most participating families received public assistance (43%) and reported food insecurity (71%). 39% of parents were single, 17% reported cohabitating, 23% were married, and 21% reported being widowed, separated, or divorced.

### Study Procedures

All study components were approved by the Pennsylvania State University institutional review board. All parents and guardians completed informed consent procedures, and adolescents assented to participate.

Participant recruitment was conducted using flyers placed at community agencies and local schools, through informational brochures and goody bag distribution at community and school events and sending adolescents home from school with informational brochures. Recruitment materials specified the purpose of the study as understanding how the Building a Strong Identity and Coping Skills (BaSICS) program can support and grow a child’s natural ability to manage stress and use the skills to improve physical and mental health. Parents were informed that participation was both voluntary and confidential and that the information that they shared would not be provided to the child’s school. Families that expressed interest were then contacted by program staff to participate in eligibility screening.

In-person pretest appointments were conducted in the adolescent’s school or the project offices located in the community in the evenings. Assessments consisted of interviews, questionnaires, and the modified Trier Social Stress Task (TSST-M; Yim et al., 2010). Families were provided $40 after completing the pretest assessment battery. Parents completed self-report and parent measures on tablets while children were concurrently completing assessment measures and procedures. Following pretest assessments, adolescents’ were randomly assigned to either the intervention or control condition using a Research Randomizer (randomizer.org), which created 24 sets of numbers (0 or 1) that were then printed out, placed in sealed envelopes, and given to the adolescents in the order generated by the Research Randomizer.

Seventy adolescents were randomly assigned to the intervention. 58 adolescents completed the 16-session BaSICS intervention in-person over 8 weeks. Fifty-nine adolescents were assigned to the control group and did not participate in the intervention. Seven groups with a mean size of 7.3 children attended an average of 12.3 sessions co-led by two lead facilitators and one assistant facilitator. Groups met for two hours twice a week. The intervention was administered in a community health center and two middle schools within the two urban neighborhoods. Two weeks after the final intervention date, participants were asked to complete a post-test assessment, with 10 weeks between pre- and post-test assessment meant to minimize the practice effects of the TSST (Petrowski et al., 2012). All participants were debriefed following the TSST at their final assessment. They were told that the confederates were not actually judging their performance and had been instructed to remain completely neutral during the procedure. Saliva samples were taken in the interview rooms.

The Posttest assessment was identical to pretest assessment. Upon completion, families received $60.

Groups were conducted at a community health center and two middle schools all of which are located in neighborhoods in which the families resided. Ninety-one parent-child dyads were recruited into the study and completed all pretest measures (62.6% of children and 91.6% of caregivers were female), but seven dropped out of the study prior to starting the intervention, leaving an analysis sample of *N* = 84. No differences between the dropped participants and those remaining in the study were evident on indices of child internalizing, parental depression, or perceived economic hardship. All of the 7 who dropped out after pre-assessment were single mothers with an average income of $8300/year in comparison to $21,000/year for the remaining dyads. These parents all reported that their schedules no longer accommodated the intervention, despite agreeing to be available during the screening process.

To enhance participation in data collection, several efforts were taken to reduce barriers, including locating intervention groups in the communities in which the families resided, providing transportation and meals, and paying in cash. To enhance retention, parents of children in the intervention group were texted weekly by one of the facilitators to briefly outline the key skills taught during the week’s sessions and to provide feedback on their child’s engagement. All families received regular newsletters containing information about the study and helpful tips on topics such as free activities available in their community and healthy eating.

Sixteen dyads did not return for their posttest assessment. Most attriters (75%) were from the control group. Hence, intervention retention was 91%, while control retention was 70%. Completers and attriters differed on CBCL (*F*(1,62) = 3.93, *p* < 0.055) and YSR total problems (*F*(1,62) = 4.0, *p* < 0.05), reflecting poorer pre-intervention functioning for completers—this was not different for intervention versus control participants, all ps > 0.10.

### Building a Strong Identity and Coping Skills (BaSICS) Intervention

The BaSICS intervention (Rajagopalan et al., 2025; Pham, Bendezú & Wadsworth, 2023; Mayo et al., 2022; Wadsworth et al., 2022; Wadsworth et al., 2022; McDonald et al., 2020) was developed based on these tenets and targets early adolescence as a fertile time for the development of coping and self-regulation. BaSICS targets two types of engagement coping, primary (e.g. problem solving, emotional expression and regulation) and secondary (e.g. cognitive restructuring, positive thinking) control coping in order to strengthen active coping with stressors and prevent and ameliorate negative long-term psychosocial outcomes. This intervention begins by improving adolescents fundamental understanding and awareness of emotions, their functions, and their impacts on physical and psychological wellbeing. In the first module, adolescents’ are taught about the physiology of stress and emotions, the various types of stressors that they might encounter (e.g. daily stress, life stress, neighborhood stress) and active ways to cope with these stressors. These coping skills aim to actively change or alter the stressor (i.e. problem-solving) or alter how adolescents think about and react to these stressors (i.e. emotional expression, positive thinking). These coping strategies have been shown to be most effective in coping with stress. Improved coping has then been hypothesized to lead to improved cortisol reactivity responses in adolescents. The second module then focuses on identity development, and empowerment through community action as additional ways of taking action in the face of PRS. Research has demonstrated identity acts as a coping resource for marginalized adolescents, and that community action is another form of active coping for these adolescents. Thus targeting identity and community action are means of broadening adolescents’ coping repertoire. Further information regarding this intervention and adolescents enrolled in the intervention can be found in (Pham et al., 2023). This intervention, then, according to the APRS, would lead to improvement in adolescents’ coping skills, and therefore, family well-being.

### Measures

#### Condition

Adolescents were randomly assigned using Research Randomizer (randomizer.org) to the intervention condition (participating in the BaSICS intervention) or control (not participating in the intervention). These intervention conditions were then coded as 0 (control) and intervention (1).

#### Response to stress questionnaire (RSQ)

Each member of the dyad completed the Response to Stress Questionnaire which aims at understanding voluntary and involuntary coping strategies used when the participant encounter stressors. This questionnaire assesses primary control engagement coping, secondary control engagement coping and disengagement coping. Responses range on a Likert scale from 0 (*not at all*) to 3 (*a lot*). Ratio scores were determined by using the sum of each factor divided by the total score (i.e., creating a proportion of their reported coping that comprised primary control as compared to total utilized coping;)These ratio scores were then used to determine their use of specific types of coping skills. These had internal consistencies that were adequate at the pre-intervention time points (Primary control (α = 0.74), secondary control (α = 0.76), and disengagement coping (α = 0.76)). This study looked at the difference in coping response at Time 1 and Time 2 by controlling for Time 1. This study used parent report of parents own primary and secondary control engagement coping.

#### Parent depression symptoms

Parents completed the Beck Depression Inventory (BDI) (Beck et al., 1961) before their children began the intervention, and again immediately following intervention. The Beck Depression Inventory is 21 item scale that has a mean internal consistency of 0.86 and has been validated across diverse samples (e.g. Garcia-Batista et al., 2018). This study looked at the difference between parent depressive symptoms at Time 1 and Time 2 by controlling for Time 1.

#### Coping

Adolescents were administered an open-ended interview using the Coping Skills Scale, which comprised of vignettes related to financial stress. They were each asked how they would respond to these stressors (e.g., not being able to afford to go to the movies with friends). Adolescents were asked how they would cope with each situation. Each coping response was coded to determine whether these strategies were primary or secondary engagement coping or disengagement coping, and specific type of coping strategy (e.g. cognitive restructuring). In this study, these measures were used to assess child acquisition of coping (e.g. STEPS, THINK) in hierarchical regressions.

#### Covariates

Parent-child dyads completed demographic information questionnaires including child sex. Additionally, cortisol reactivity was measured via saliva collected via passive drool into a 5-millimiter tube during the TSST-M. Children completed the sample immediately prior to beginning the TSST-M (T1), and immediately after completing the task (T2). Samples were then stored in a −80 °C freezer at the Pennsylvania State University. The mean intra-assay and inter-assay coefficients of variation for cortisol are 4.60 and 6.00%, respectively. For alpha-amylase, intra-assay and inter-assay coefficients of variation are 5.47 and 4.70%.

#### Intervention fidelity

Several strategies were used to establish and maintain provider skills and to ensure fidelity to the intervention material. First, the program has a detailed, step-by-step, session-by-session intervention manual as well as a behavior management manual. Facilitators were trained by the intervention developer and conducted their first intervention groups alongside an experienced lead facilitator to provide modeling of intervention delivery. Second, a licensed clinical psychologist versed in the intervention conducted weekly supervision with facilitators to review past week’s content and preview upcoming content. Adherence to protocol was monitored via coding of videotapes of sessions and in-session raters. As rated by at least two raters per session, 89.9% of required elements were covered. Inter-rater agreement was κ = 95%.

### Analysis Plan

Simple mediation analyses were performed in (IBM Corp., 2010) SPSS (version 29.0) using the Process Macro Plug-In version 19 (Hayes, A.F., 2022). Multiple linear regression analyses were run to further explore intervention effects for children’s coping affecting parent coping and depression. Missing data were deleted listwise.

## Results

### Descriptives and Correlations

Pre-intervention and post-intervention engagement of parent-reported parent primary control coping were significantly positively associated with each other (*r* = 0.268, *p* < 0.05). Cortisol reactivity at pre-intervention and post-intervention were also significantly positively associated (*r* = 0.383, *p* < 0.01), as were parent BDI scores at pre and post-intervention (*r* = 0.590, *p* < 0.01). Parent reported depression pre-intervention was significantly negatively associated with parent engagement of primary control coping at pre-intervention assessments, wherein parents with higher primary control coping had fewer reported symptoms of depression on the BDI (*r* = −0.285, *p* < 0.01). Additionally, parent BDI scores post-intervention were negatively associated with parent primary control coping at post-intervention, with a higher score predicting decrease in reported primary control coping (*r* = −0.334, *p* < 0.01). Parent primary control coping at time one was also significantly correlated with parent report of child coping at time 1 (*r* = 0.476, *p* *<* *0.01*). Parent primary control coping was significantly associated with parent report of child primary control coping at time two (*r* = 0.445 *p* < *0.01*) (Tables 1, 2).

**Table 1:** Means, Standard Deviations and Correlations

Variable	1	2	3	4	5	6	M(SD)
1. Cortisol Reactivity (Time 1) (child)	—						−0.53(2.37)
2. Cortisol Reactivity (Time 2) (child)	0.383**	—					−0.30(2.89)
3. BDI Sum Total (Time 1) (parent)	0.043	−0.028	—				12.25(12.43)
4. BDI Sum Total (Time 2) (parent)	0.031	−0.254	0.590**	—			11.76(11.93)
5. Primary Control Coping (Time 1) (child)	−0.003	−0.057	−0.285**	−0.182	—		0.19(0.04)
6. Primary Control Coping (Time 2) (child)	0.076	0.129	−0.106	−0.334**	0.268*	—	0.19(0.04)

**Table 2:** Correlation between Parent and Child Primary Control Coping

Variable	1	2	3	4
1. Parent Primary Control Coping (T1)	—			
2. Parent Report Child Primary Control Coping (T1)	0.476**	—		
3. Parent Primary Control Coping (T2)	0.268*	0.216	—	
4. Parent Report Child Primary Control Coping (T2)	0.475**	0.580**	0.445**	—

### Mediating Effects of Parent Coping

**Fig. 2:**
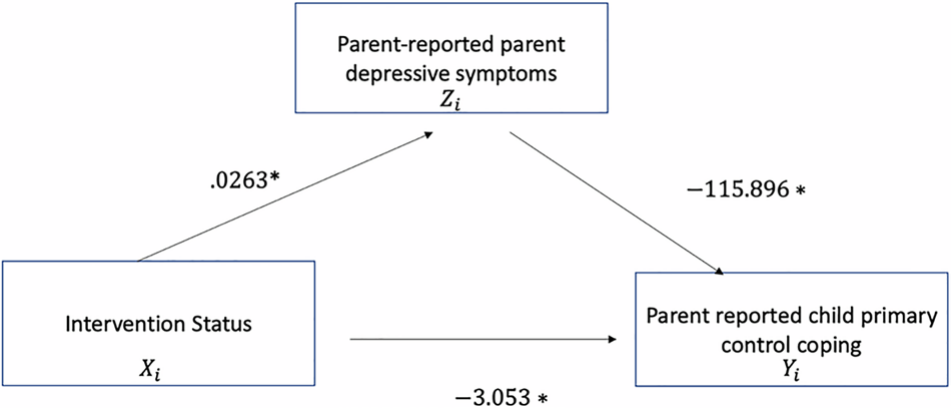
Mediation Model

Controlling for the sex and cortisol reactivity of the child participants, the mediating role of parental engagement of primary control coping was explored using Model 19 of the plug-in PROCESS macro in SPSS developed by Hayes (2022). Figure 2 shows the mediation analysis results which provide evidence that child participation in an intervention targeting coping skills is directly associated with a decrease in parent depressive symptoms from beginning the intervention and post-intervention. The results showed that there was no significant total effect between child intervention status and parent mental health (*B* = 4.01, *p* = 0.206). However, research has shown that mediation effects can still be present without a total effect (O’Rourke & MacKinnon, 2018). There was evidence of a significant direct effect of intervention status on parent depression scores (*B* = 7.07, *p* = 0.025). Additionally, child intervention status significantly predicted an increase in parent reported parent engagement of primary control coping skills (*α* = 0.0263). Additionally, this change in parent reported parent use of primary control coping skills was also associated with a decrease in parent reported depressive symptoms after intervention(*b* = −115.896). The mediating effect of parent coping was tested and yielded significant results (*ab* = −3.0531) based on a mediation model run using 5,000 bootstrap resamples entirely above zero (95% Confidence Interval = −6.289, −0.419). This supports the hypothesis that the relationship between participation in a coping intervention and changes in parent depression scores is significantly mediated by increased use of primary control coping by parents. An additional mediation model was run to analyze whether secondary control coping significantly mediated the relationship between adolescents participation in a coping intervention and changes in parent depression scores, however, the total effect (*B* = 3.31, *p* = 0.29) and mediating relationship were both insignificant (95% Confidence Interval = −3.69, 1.51).

### Possible Mechanisms Explaining the Mediating Effects of Parent Coping

Once again controlling for sex of child participants, regression analyses were run to probe the possible mechanisms by which this mediating relationship might exist. Table 3 shows the results of these regression analyses. The tests revealed that participation in a coping intervention (intervention status), cortisol reactivity changes, child engagement of problem solving, and parent engagement of secondary control coping, predicted 48.8% of the variance in changes in parent depression scores on the Beck Depression Inventory, ${R}^{2}$ = 0.488, *F* (4, 42) = 4.99, *p* = 0.002. The data suggest that changes in parent engagement of secondary control coping skills was also a significantly associated with fewer reported depressive symptoms, though not as a mediator, but as a promoter of changes, β = −87.301, *t* = −2.230, *p* = 0.031. Cortisol reactivity changes and child problem solving were not found to be significant predictors of this relationship.

**Table 3:** Hierarchicical Regressions

Variable	B		SE B	β			B	SE B	β
Constant	7.55		11.26				29.02	10.96	
Intervention Status	4.23		2.95	0.191			2.29	3.63	0.104
Sex	−0.92		3.2	−0.039			−4.04	2.87	−0.173
Primary Control Coping (T1)	6.91		37.99	0.025			22.55	36.52	0.08
BDI Sum Total (T1)	0.41		0.13	0.44			0.42	0.12	0.45
Cortisol Reactivity (T1)	−0.041		0.54	−0.01			0.92	0.57	0.23
Secondary Control Coping (T1)							10.85	31.71	0.04
Primary Control Coping (T2)							−45.09	42.81	−0.16
Secondary Control Coping (T2)							−87.30	39.15	−0.34
Problem Solving STEPS (T2)							1.22	0.97	0.20
Cortisol Reactivity (T2)							−0.97	0.56	−0.23
R^2^	0.25						0.49		
F for change in R^2^	2.48*						4.99**		

## Discussion

This study sought to examine the effects of a child-focused stress and coping intervention on caregiver coping and mental health outcomes. Specifically, we examined the extent to which caregivers of adolescents’ enrolled in a coping intervention (BaSICS) displayed improvements in effective coping and mental health post-intervention. We hypothesized that parents of adolescents enrolled in BaSICS would report fewer depressive symptoms on the BDI, and that this relationship would be mediated by their own engagement of primary control coping skills. Further, we investigated mechanisms by which this relationship might be occurring. We found these caregivers reported less BDI depressive symptoms immediately following intervention. Additionally, we found that this relationship was mediated by parents increased use of primary coping skills post-intervention. Additional analyses revealed that increases in parent engagement of secondary control coping also predicted improved parent depressive symptoms. Our analyses did not demonstrate an association between cortisol reactivity changes in adolescent and parental depression. Similarly, there was no significant association between the engagement of problem solving by adolescent and parental depressive symptoms post-intervention.

These findings suggest that BaSICS, an intervention that has demonstrated significant and sustained improvements in child coping (McDonald et al., 2020; Wadsworth et al., 2022), promotes more effective coping in parents as well. Additionally, consistent with our hypothesis, increased parental coping post-intervention was associated with a decrease in depressive symptoms in parents. Parent-reported primary coping mediated the relationship between intervention status (i.e. whether the child received the intervention or did not) and changes in parent BDI scores. Given the significance of this initial mediation model, we explored additional mechanisms by which this relationship exists through a multiple regression model, including changes in HPA reactivity, child problem solving (STEPS), and parent secondary control coping on parent reported depressive symptoms. Secondary control coping (e.g. positive thinking, cognitive restructuring,) was also associated with a decrease in depressive symptoms in caregivers whose children were enrolled in BaSICS.

Research has consistently shown that families with limited financial resources tend to use fewer primary and secondary control coping compared to families from higher socioeconomic backgrounds. Additionally, these families often report a greater reliance on disengagement coping (i.e. avoidance) skills. These coping differences may be related to a number of factors. For instance, relying on disengagement coping may be due to feelings of powerlessness, lacking resources of opportunity or feelings of low self-efficacy to use these skills. Additionally, the uncontrollable nature of the stressors may render primary and secondary control coping less useful, ineffective or difficult to engage. Our results highlight how teaching adolescents these skills (e.g., Pham et al., 2023; Wadsworth et al., 2011; McDonald et al., 2020) may spillover into the family system. As adolescents are better able to cope with daily and life stressors, and experience improvements in mental and behavioral health, the family system also experiences positive improvements and better outcomes.

To date, the extant literature has focused on the relationship between caregiver stress and mental health and its subsequent impacts on child coping. However, the APRS posits that child and caregiver coping, health, and stress are bidirectional in nature. Cappa et al., (2011) provided initial support for the bidirectional relationship between parent and child coping in a sample of caregivers enrolled in a parenting program and child coping. They found that children with parents reporting higher stress tended to have more difficulty coping with academic and social challenges than children with parents reporting lower overall stress. To our knowledge, no research has yet looked at the impacts of a child-focused intervention on parent mental health and coping. Our findings suggest that adolescent enrollment in an intervention focused on teaching them primary control coping skills was significantly related to reduction in their caregiver’s depressive symptoms. Further, the relationship between adolescent intervention status and caregiver depressive symptoms is explained by caregiver use of primary control coping skills. Caregivers of adolescents enrolled in the coping intervention tended to increase their use of primary coping skills following the intervention and, in turn, tended to experience fewer depressive symptoms. This relationship may be explained by multiple factors.

First, as elucidated by the Adaptation to Poverty Related Stress model, coping does not exist in a vacuum. Instead, it is embedded within the family system and relies on the dyadic caregiver-child relationship, such that as a child’s ability to cope with uncontrollable stress increases or decreases, so too does their caregivers ability to cope (Wadsworth et al., 2011). Wadsworth and colleagues, (McDonald et al., 2020, Pham et al., 2023) demonstrated that participation in BaSICS, an intervention designed to target primary and secondary control coping skills in adolescents, was significantly associated with improvement in adolescents coping repertoire, such that adolescents enrolled in BaSICS utilized more primary and secondary control coping skills than adolescents not enrolled in the intervention.

It may be that while enrolled in this intervention, as children gained greater knowledge of active coping skills (e.g. primary control and secondary control coping skills), they practiced these in their daily lives with greater frequency (Pham et al., 2023; Wadsworth et al., 2011; McDonald et al., 2020; Pham et al., 2023), leading to a decrease in internalizing/externalizing symptoms. Parents may then both learn these coping skills from their children, and begin practicing them as well, as shown by increased report of primary and secondary control coping by both parents and adolescents. Following the greater use of primary and secondary control coping, parents themselves may begin to experience a decrease in depressive symptoms given the demonstrated relationship between engagement of appropriate coping skills and improved mental health. This is demonstrated by the significant correlation between caregiver primary control coping and caregiver-reported adolescent primary control coping at pre-intervention and post-intervention, indicating that a caregiver’s ability to cope with stressors and an adolescent’s ability to cope with stressors are related, and that, as a youth coping repertoire increases, so too does parental engagement of primary and secondary control coping skills.

Additionally, in a study of adolescents aged 10–16 and their parents, adolescents with reported social support (i.e. parental/adult support) demonstrated more effective coping through social support seeking (seekng safety, seeking help, problem-solving) and individual coping strategies (self-expression, self-soothing, self-care and avoidance) than those who reported little to no adult support. For those adolescents with no adult support, many coping strategies such as distraction, problem-solving and self-care, actually exacerbated the relationship between psychological symptoms and stress exposure, and only avoidance was an effective coping strategy (Reife, Duffy & Grant, 2020). This might indicate that the family structure and dyadic nature of coping and stress management is of the utmost important to the utilization of effective coping strategy. That is, despite adolescents learning coping skills, they may not be able to engage them appropriately without the support of adult caregivers, and through enrollment in the BaSICS intervention, may have a better avenue of engaging dyadic coping or prompting the use of helpful coping strategies. For these adolescents and their families, BaSICS and adolescent-focused interventions may provide a method of ensuring more intentional use of coping skills.

Second, research has demonstrated that coping is strongly related to mental health outcomes in both adolescents and adults. Kim, Neuendorf, Bianco & Evans (2016) found that coping, in particular disengagement coping, in adolescents aged 13–17 helps to elucidate the relationship between chronic exposure to childhood poverty and mental health outcomes. Further, the present study suggests that, as children learn new coping skills and ways to implement them, caregivers are better able to utilize their own coping skills. Prior studies have shown that child behavioral challenges are associated with caregiver mental health difficulties (Riahi, 2022), specifically with depressive symptoms (Narayanan & Naerde, 2016; Bagner et al., 2013). As children are better able to use primary control coping skills, behavioral difficulties may be alleviated, leading to a decrease in caregiver depressive symptoms. Further, as caregivers are more able to engage primary control coping skills, they may experience greater feelings of self-efficacy in their ability to control, change, and/or cope with poverty-related stress. This greater sense of self-efficacy may then improve caregiver depressive symptoms. Greater engagement in these coping strategies not only helps caregivers manage poverty-related stress, but also compounded stressors that often co-occur with poverty-related stress (e.g. marital strain, parenting stress, job stress). This decrease in stress may also extend available coping resources more broadly, such that parents feel able to use engagement coping skills (e.g. problem solving, cognitive restructuring) in response to various daily-life stressors. Given adolescent involvement in this intervention, they may be actively and passively teaching their caregivers these useful coping strategies. At the same time, adolescent improvements in behavior and mental health post-intervention, may broaden parents ability to cope with their stress due to decreased parenting stress. Additionally, Wadsworth et al., (2011) found that primary control coping acted as a mediator between economic hardship and internalizing problems in caregivers and their children. Thus, caregivers, following their adolescent’s participation in BaSICS, may begin to utilize coping strategies with greater frequency and more effectively, and thus, have greater ability to improve their surroundings, cope with uncontrollable factors, and subsequently, improve internalzing symptoms such as depressive symptoms.

Adolescent mental health has been shown to impact caregiver coping via child physical health (i.e. child chronic illness). In pediatric populations, the parents of children with both physical and mental health challenges have been shown to have more difficulty using effective coping strategies (Vernhet et al., 2019). As such, with these associated improvements in child mental health and child behavior through BaSICS, parents may find greater ability and opportunity to use primary control coping skills (McDonaldet al. 2020, Pham et al., 2023) and, further, improve parent mental health. This suggests that as child behavior improves, the compounded stress of caregiving would decrease, leading to decreases in caregiver depressive symptoms.

In the present study, simple regression analyses did not reveal an association between child engagement of problem solving or cortisol reactivity changes for adolescents, with parental BDI scores. Problem solving is one of many primary control coping strategies that is taught to adolescents during the BaSICS intervention. Though prior research (McDonald et al., 2020; Pham et al., 2023) demonstrated that adolescents acquire the problem-solving skills during intervention, problem solving alone as a coping skill may not be the only mechanism by which a relationship exists between adolescent intervention status and caregiver mental health. Adolescents are taught many types of coping strategies throughout the intervention, including problem solving, cognitive restructuring, mindfulness, and biofeedback. Further, adolescent participate in modules that target racial and ethnic identity and social action. Thus, the effects of the intervention as evidenced by our study may be due to multiple, if not all, of these various skills and the adolescents psychosocial and behavioral improvements. Future work should explore the ways in which modules revolving around cultural, racial, and ethnic identity may also impact parent and adolescent coping following intervention.

## Strengths and Limitations

This study should be understood in light of several strengths and limitations. First, the study followed a sample population that identified as majority non-White. These populations are largely underrepresented in research and are representative of populations greatly impacted by uncontrollable stress such as poverty-related stress. Given this, studies that provide greater insight into family functioning in the face of stress and means of improving family outcomes (i.e. interventions) are of significant importance.

These findings may be somewhat limited due to several factors. Of note, the sample size of our study is small. Given this, the study should be interpreted with caution of its generalizability. Though also a strength, the majority of our sample identify as Black or African-American, and exploration into other racial and ethnic groups is warranted for a greater understanding of the relationship between child coping and parent well-being. Future directions would include replications of this study with a larger sample and an exploration into the impacts of a child-focused coping intervention on parent well-being in a more diverse sample.

Additionally, we examined the changes of coping and parent depression at two timepoints, prior to intervention and immediately following the intervention. Given there was a significant number of families lost to follow-up owing in part to COVID (Fig. 1), further research may aim to look at the lasting impacts of a child-focused coping intervention on adolescents and their families, and how long effects persist following intervention.

Lastly, our study examined parent depressive symptoms as a means of understanding one facet of parental well-being prior to and following an adolescent-facing coping intervention. However, there are multiple facets of well-being, including, reports of stress, physical well-being, and anxiety symptoms. Future studies might examine more facets of parental well-being in response to interventions like BaSICS.

## Conclusion and Clinical Implications

This study aimed to establish whether enrollment in a child-focused coping intervention was related to improved parental depression. Specifically, it assessed the mediating relationship of increased parental primary control coping on parent depressive symptoms following the intervention for their children. The results of this investigation were in accordance with our hypotheses and suggest an existing mediation for parents of adolescents participating in BaSICS, of the relation between parental primary control coping and parental depression. Additionally, we explored the possibility of other variables that might be partially responsible for this relationship and found a significant association of parental secondary control coping. These findings enhance our understanding of the importance of intervention for adolescents facing uncontrollable stress, and, even greater, the ways in which coping is consequential to family functioning and systems. Given our knowledge of the importance of early adolescence as sensitive period for change and development in adolescents, more interventions and services targeting adolescents coping at this time may be leveraged to improve not just adolescents’ developmental outcomes, but the wellbeing of caregivers and families. Interventions aimed at adolescent functioning, thus, are not just impacting the long-term health of adolescents, but of caregivers, and the systems in which they exist. This analysis lays the groundwork for future research into widespread family impact of adolescent-based interventions, and, possibly, family-based interventions. Prior research demonstrated the far-reaching consequences of stress within families and communities, however, little literature prior to this study has strived to understand the utility of coping interventions within the home. Potential future clinical directions may include a module or section involving children actively teaching parents the new coping skills they have learned, enhancing the “trickle-down” effect of the intervention. Parents whose adolescents are enrolled in coping interventions such as BaSICS may benefit from actively engaging their adolescents in a discussion or demonstration of what they have learned, not only to reinforce the information for the adolescents, but to learn themselves. Prompting of the use of active engagement coping and dyadic participation in strategies (e.g. cognitive restructuring, problem solving) may be a fruitful avenue for the continued improvement of parent mental health following intervention. Additionally, as policy and intervention are developed, the knowledge that not only are the challenges associated with poverty related stress, lasting mental health impacts, and interactional difficulties dyadic, the family system is permeable and dynamic, and the introduction of positive interactional patterns, internal motivation and ability to actively cope, can cause widespread positive changes on the family system. Family support services may then begin recommending not only parenting skills support, but child coping skills support, therapeutic services and interventions with the intention of targeting the family system across the different contributors and allowing for the greatest combined resource utilization. Often, the burden of coping with and managing uncontrollable stress falls on caregivers. However, given the dyadic nature of coping, and the dyadic transmission of mental health difficulties, consideration of the impact of chronic stress on adolescents may allow for multiple points of intervention and better outcomes. Thus, future interventions may conceptualize changes not solely from an individualistic lens (i.e. teach adolescents coping skills to improve adolescent well-being alone) to a more family-systems lens, and begin to better target the systems as a whole rather than each member of the system.

This study demonstrates the ability of adolescent-focused interventions to integrate active coping throughout the family system, improving mental health outcomes for caregivers. BaSICS has previously been shown to be efficacious in improving mental health outcomes for adolescents’ as well, and taken in conjunction with this study, the clinical implications are far reaching. Interventions aimed at targeting adolescent coping can impact family systems, and adolescents’ own wellbeing and stress management has positive ramifications for caregivers within their systems. Through the dyadic nature of coping, interventions have the ability to disrupt the negative force of uncontrollable stress on an entire family system and adolescents’ serve as an optimal point of intervention.
